# Pre-operative planning for reverse shoulder arthroplasty in low-resource centres: A modified Delphi study in South Africa

**DOI:** 10.1051/sicotj/2024021

**Published:** 2024-05-30

**Authors:** Pududu Archie Rachuene, Roopam Dey, Ntambue Jimmy Kauta, Sudesh Sivarasu, Jean-Pierre du Plessis, Stephen Roche, Basil Vrettos

**Affiliations:** 1 Department of Surgery, Division of Orthopaedic Surgery, Groote Schuur Hospital Cape Town South Africa; 2 Department of Orthopaedics, Steve Biko Academic Hospital, University of Pretoria Pretoria South Africa; 3 Department of Human Biology, Division of Biomedical Engineering, University of Cape Town South Africa; 4 Biomedical Engineering Research Centre (BMERC), University of Cape Town South Africa; 5 Health through Physical Activity Lifestyle and Sports (HPALS), University of Cape Town South Africa

**Keywords:** Reverse shoulder arthroplasty, Preoperative planning, Low-resourced practice

## Abstract

*Background*: Pre-operative planning for reverse shoulder arthroplasty (RSA) poses challenges, particularly when dealing with glenoid bone loss. This modified Delphi study aimed to assess expert consensus on RSA planning processes and rationale, specifically targeting low-resourced institutions. Our objective was to offer pre-operative decision-making algorithms tailored for surgeons practising in resource-constrained hospitals with limited access to computed tomography (CT) scans. *Methods*: A working group generated statements on pre-operative imaging and glenoid of glenoid morphology and intra-operative decision-making. The study was conducted in three stages, with virtual consensus meetings in between. Stages 2 and 3 consisted only of closed questions/statements. The statements with over 70% were considered consensus achieved and those with less than 10% were considered disagreement consensus achieved. *Results*: Twelve shoulder surgeons participated, with 67% having over five years of experience in shoulder arthroplasty. In the absence of glenoid bone loss, the sole use of plain radiographs for pre-operative planning reached consensus and is recommended by these groups, while 100% advise using CT scans when bone loss is present. Most surgeons (70%) recommend using patient-specific instrumentation (PSI) in cases of structural bone loss. Most of the statements on intra-operative decision-making related to component placement and enhancing stability failed to reach consensus. *Conclusion*: While consensus was achieved on most aspects of pre-operative imaging and planning, technical aspects of surgery lacked consensus. Planning for patients with structural glenoid bone loss necessitates CT scans and planning tools.

## Introduction

Glenoid wear and structural defects necessitating intervention have been documented in approximately 15% of shoulders undergoing arthroplasty [1]. Effective preoperative planning is challenging and crucial to enhance procedural efficacy and optimize surgical outcomes in patients undergoing reverse shoulder arthroplasty (RSA) [2]. The first steps of planning shoulder arthroplasty encompass patient history taking, clinical examination and plain X-rays with Grashey views, however, computed tomography (CT) scan with 3D reconstructions are recommended for evaluation of glenoid morphology [3]. Failure to recognize bone defects and morphological alterations during planning may lead to intra-operative challenges and early component failure, with technical errors and failure to identify glenoid alterations being common contributors [4].

While CT scans are advocated as the optimal imaging modality for RSA pre-operative planning due to their superior ability to define the extent of glenoid wear and morphological changes [5], their availability is limited in many low- and middle-income countries (LMICs) [6]. X-rays continue to be the most used and widely accessible modality. Although computer software planning tools have demonstrated accuracy in guiding prosthesis selection and placement, they are dependent on the availability of CT scans and have certain limitations [7, 8]. Notably, there is a lack of consensus or guidelines for pre-operative planning before RSA in resource-constrained settings, particularly for shoulders with glenoid bone loss.

In response to the highlighted obstacles, this study aimed to provide guidance and devise an algorithm for shoulder surgeons in LMICs on RSA pre-operative planning procedures. Key aspects investigated included the selection of imaging modalities, the utilization of 3D reconstructed CT images, planning tools, and component placement strategies. We are not aware of an existing consensus study on this subject.

## Materials and methods

### Study design

The study aimed to establish consensus on pre-operative planning and intra-operative protocols for reverse shoulder arthroplasty in patients with glenohumeral joint arthritis, particularly in resource-limited settings. A working group comprising five shoulder surgeons (PAR, SR, NJK, JPD, and BV) employed modified Delphi techniques to develop consensus statements. These statements were categorized into pre-operative planning procedures, glenoid component placement, and participant experiences.

### Consensus participants, recruitment processes and follow-up

Fourteen experts from diverse hospital settings in South Africa were invited to participate, with twelve from the Western Cape Province. The majority of participants (67%) possessed over 5 years of experience in shoulder surgery, with 42% having more than a decade of experience. Regarding resources, 80% reported unlimited access to CT scans, while only 50% had unlimited access to computer software planning tools. Conversely, only 20% reported unlimited access to patient-specific instrumentation (PSI).

**Figure 1:**
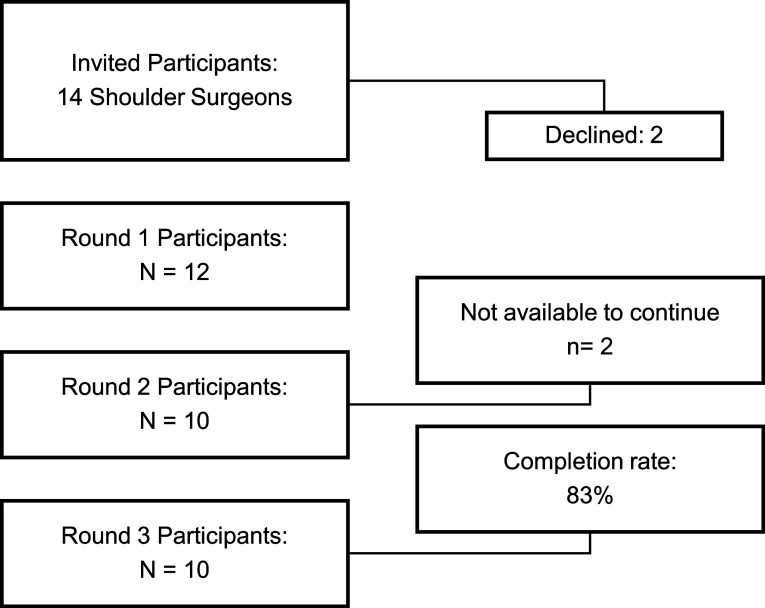
A flowchart depicting the recruitment process.

Recruitment processes are illustrated in Figure 1. In phase 2, two participants withdrew, resulting in a completion rate of 83% with 10 participants successfully completing all three phases. By the conclusion of phase 2, agreement was reached on 15 out of 31 statements, accounting for 48% of the total. These agreements were characterized by over 70% consensus and less than 30% disagreement. Out of the 16 statements that lacked consensus, 9 of them were refined and attained consensus in phase 3, resulting in a consensus rate of 81%.

### Establishment of consensus processes

The study encompassed three stages conducted between April 2020 and October 2022, with a minimum six-month gap between each phase. Initial phases included both open-ended and closed-ended questions, while subsequent phases focused on closed-ended statements. Virtual meetings were held with the working group and participants to evaluate responses, categorize statements, and determine consensus levels. Statements with 70% or more agreement were considered consensus, while those with less than 30% agreement were discarded. Feedback and comments from participants were incorporated into subsequent phases.

## Results

### Pre-operative imaging and glenoid morphology evaluation

The use of Grashey and axillary views radiographs as an initial imaging modality for planning for RSA reached a strong consensus (90%). Contralateral shoulder imaging for comparison purposes provided the disease is unilateral also reached a consensus. Although routine use of CT scans and planning tools did not reach a consensus, with only 60% agreement, plain X-rays were considered to be unreliable for the determination of glenoid morphology alterations. Notably, all surgeons agreed (60% “agree”, 40% “strongly agree”) on CT scans and planning software necessity when structural glenoid defects were present. Seventy per cent of surgeons recommended manual measurement on 2D and 3D CT scans alongside planning tool measurements, expressing concerns about planning tool accuracy (50%). Additionally, 2D CT scans were favoured over conventional radiographs for quantifying glenoid measurements (80% consensus), with plain X-rays deemed unreliable for this purpose (90% agreement). The routine use of 3D printing in RSA was unanimously discouraged (100% disagreement). Table 1 provides a summary of consensus and recommendations regarding preoperative imaging.

**Table 1:** Key consensus recommendations on preoperative imaging.

Modalities	Surgical planning and execution recommendations
Plain radiographs	Employ anteroposterior, Scapula-Y, axillary, and Grashey views plain X-rays routinely for initial assessment of glenoid wear. Avoid relying solely on these views for quantifying glenoid bone loss or morphological alteration.
CT scan (2D and 3D)	Regularly utilize in all cases where significant glenoid wear is evident on plain X-rays. Always conduct manual measurement of glenoid bone loss. If CT scan imaging is not feasible, refer to a facility equipped with CT scan capabilities or postpone surgery until CT scan is accessible.
Computer software planning tool	Recommended for cases with structural or significant glenoid wear and/or notable morphological changes. Do not solely depend on automated glenoid native measurements; cross-reference with manual measurements for accuracy.
3D printing of the Scapula model	No recommendation for or against use.

### Perioperative decision making

A consensus on glenoid baseplate placement parameters was not reached. Although 70% of participants accept neutral or anteverted components, only 60% accept 10° and 10% 15°. Eighty per cent accepted an inferior slanted component, but the extent was unclear. Nobody accepted a superiorly tilted component. The accuracy of the base of the coracoid as a reference point for assessing glenoid wear and joint medialization during surgery was agreed upon by 70% of the respondents, with 30% strongly agreeing. Walch classification was not recommended for use in component selection and placement. Routine PSI use was opposed by 70% of participants. When structural faults were present, 60% of respondents agreed and 10% strongly agreed to employ PSI.

Consensus was reached on the number of screws, with 70% of participants choosing 2 additional screws to the middle peg/screw. Central screw/peg penetration into the native bone and required baseplate support by the native bone were not agreed upon. However, most surgeons (60%) consider 75% centre peg penetration into natives as stable. About 40% chose 80% penetration into the native bone, 30% chose 50%, and 30% chose a bicortical screw as a stable construct when using a component with a central screw. No consensus existed on baseplate contact with native bone for stability. The majority (50%) would accept 80% baseplate contact with native glenoid, whereas 40% would accept 50%. The Delphi recommendations regarding perioperative processes are condensed in Table 2.

**Table 2:** Summary of perioperative recommendations outlined from the consensus statements.

Decision making	Use the base of coracoid as a dependable landmark to assess joint line medialization. Remain flexible to adjust plans during the perioperative phase.
Patient specific instrumentation	Consider utilizing for glenoids with structural or substantial wear, or noticeable morphological alterations to improve baseplate positioning and fixation.
Glenoid component placement	Target: At least two peripheral baseplate screws Neutral or inferior tilt Neutral version or anteversion

Although significant effort was dedicated to pre-operative planning, all participants admitted to deviating from their initial plans during surgery. Seventy per cent estimated their deviations to be around 25% of the time, while 10% reported deviations ranging from 75% to 100%.

## Discussions

Preoperative planning for reverse shoulder arthroplasty in arthritic shoulders with morphological alterations presents significant challenges [9]. Our previously published narrative review emphasized the importance of using CT scans and computer software planning tools for this purpose, as advocated in the literature. This underscores the limitations of plain X-rays in accurately assessing glenoid morphological alterations [10]. Nevertheless, access to CT scans is still restricted in low- and middle-income countries (LMICs), despite the increasing global demand for shoulder arthroplasty surgery [6, 11]. This study evaluated consensus on pre-operative imaging and planning for reverse shoulder arthroplasty and perioperative glenoid component placement parameters in resource-constrained countries, with limited access to CT scans. Overall, consensus was reached in pre-operative imaging processes, glenoid morphology evaluation procedures and preoperative decision-making. The perioperative process failed to reach a consensus on most of the statements.

It is however important to note that this Delphi technique is not without limitations. Although the participants are experienced in performing this procedure, there is a variety of prostheses in the market, using various techniques for implantation. Nonetheless, the principles of the procedure remain the same and this should allow for a universally acceptable consensus. The Delphi technique may allow for bias due to anonymous responses and the resultant lack of responsibility for the responses given, however, this study allowed for open questions in the first phase and a virtual meeting in between the phases to ensure the inclusivity of expert opinions, reliability of responses and commitment towards the responses given.

Reverse shoulder arthroplasty stands as the preferred treatment for various challenging shoulder conditions in elderly individuals, encompassing cuff tear arthropathy, glenohumeral arthritis and complex proximal humerus fractures, among others [12]. These conditions often entail notable glenoid bone loss, requiring advanced pre-operative imaging and defect management with metal augments or bone grafts. They are associated with an increased risk of procedure failure. Failure to address these defects and achieve stable glenoid component implantation is deemed a contraindication to a single-stage RSA [10]. Contrary to current evidence, our study found a consensus favouring the primary use of plain X-rays over CT scans for pre-operative planning, with routine CT scan utilization failing to reach consensus. However, research by Dekker et al. underscores the importance of routine CT scans for total shoulder arthroplasty planning, as they enhance the assessment and measurement of glenoid morphological changes and facilitate the detection of glenoid cysts and bone loss [9]. Nonetheless, resource constraints and limited imaging equipment, particularly in low- and middle-income countries (LMICs) like ours, present significant challenges. LMICs reportedly have less than one CT scanner per million inhabitants, a stark contrast to the nearly forty scanners per million inhabitants in high-income countries (HICs) [4]. The use of contralateral X-rays for comparative purposes is recommended by these experts and supported by the literature for patients with unilateral disease, in the absence of the CT scan. Although there is no available evidence to support this recommendation, Verhaegen et al. observed that the anatomical parameters of the scapula and glenoid are symmetrical in healthy shoulder CT scan images, which supports this recommendation [13].

In our study, the surgeons recommend the use of CT scans and computer software planning tools only when planning for shoulders with structural glenoid bone loss evident on plain radiographs. The use CT scans with 3D reconstruction has been demonstrated to enhance the assessment of glenoid wear and morphology in arthritic shoulders. Plain X-rays are considered inferior to CT scans due to their tendency to overestimate the degree of wear and failure to detect bone loss [14, 15]. With reference to the native glenoid version, glenoid inclination, and wear measurement, manual methods are recommended by 80% of the surgeons who took part in our study. In planning for this procedure, it is evident from the literature that measurements on standard X-rays and automated and/or semi-automated measurements provided by the planning tools should not be relied upon in isolation [7, 8].

In the absence of infection, RSA fails due to glenoid component instability, impingement, or scapula spine fractures among other causes [16]. These causes are mainly related to technical errors during surgery and the inability to achieve component stability [17]. The use of PSI has been reported in the literature to improve the position of the glenoid component [18]. However, some studies report conflicting evidence on their accuracy [19]. Our study achieved consensus on using PSI for cases with structural bone loss, but routine adoption was not agreed upon. Existing literature supports the efficacy of planning tools and PSI in ensuring accurate glenoid component sizes and placement, thus enhancing component stability by facilitating additional and long baseplate screws [20, 21]. However, Navarro et al. (2023) found that utilizing CT scans for preoperative planning with PSI did not decrease the incidence of revision surgeries or the overall risk of complications in anatomic and reverse shoulder arthroplasty procedures [22].

With reference to glenoid component implantation, most of the participants prefer a component with a neutral version (70%) and inferior inclination (80%), however, we cannot report consensus on the degree of inferior inclination. Biomechanics and clinical studies have shown that less than 15° inferior tilt has no significant impact on range of motion and risk of notching and a tilt of 15° improves baseplate stability by increasing compression forces [23–25]. Achieving glenoid component stability is also dependent on the baseplate contact with native bone, the number and length of the screws and/or centre peg [26, 27]. The design and sizes of the baseplates and centre peg/screw design available in the market vary and this may have an impact on these parameters. In this study, there was no consensus on the centre peg or centre screw penetration but the use of 2 screws and the ability to achieve 50% baseplate to native bone contact reached consensus as some of the requirements for stable components. The clinical and biomechanical studies also seem to favour these parameters, with lower risks of failure and revision rates [28–30]. The discovery that surgeons frequently veered from their preoperative plan during the perioperative phase underscores the critical role of expertise and clinical judgment in surgical execution.

## Conclusions

In resource-constrained settings, this consensus group recommends CT scans and planning tools for obvious structural bone loss on conventional 4-view plain radiographs. Manually measuring bone loss on 2D slices should be part of bone defect planning. These results should assist a surgeon with limited resources in determining when to refer a patient for CT imaging prior to RSA. The use of 3D printing is not recommended by these surgeons. Where resources are limited, patients should be sent to a facility that has a CT scanner for pre-operative planning.

## Data Availability

The datasets used and analyzed during the current study are available from the corresponding author on reasonable request.
